# Balanced, bi-planar magnetic field and field gradient coils for field compensation in wearable magnetoencephalography

**DOI:** 10.1038/s41598-019-50697-w

**Published:** 2019-10-02

**Authors:** Niall Holmes, Tim M. Tierney, James Leggett, Elena Boto, Stephanie Mellor, Gillian Roberts, Ryan M. Hill, Vishal Shah, Gareth R. Barnes, Matthew J. Brookes, Richard Bowtell

**Affiliations:** 1 Sir Peter Mansfield Imaging Centre, School of Physics and Astronomy, University of Nottingham, Nottingham, NG7 2RD UK; 2 Wellcome Centre for Human Neuroimaging, Institute of Neurology, University College London, 12 Queen Square, London, WC1N 3AR UK; 3 QuSpin Inc., 331 South 104th Street, Suite 130, Louisville, CO 80027 USA

**Keywords:** Neuroscience, Applied physics

## Abstract

To allow wearable magnetoencephalography (MEG) recordings to be made on unconstrained subjects the spatially inhomogeneous remnant magnetic field inside the magnetically shielded room (MSR) must be nulled. Previously, a large bi-planar coil system which produces uniform fields and field gradients was used for this purpose. Its construction presented a significant challenge, six distinct coils were wound on two 1.6 × 1.6 m^2^ planes. Here, we exploit shared coil symmetries to produce coils simultaneously optimised to generate homogenous fields and gradients. We show nulling performance comparable to that of a six-coil system is achieved with this three-coil system, decreasing the strongest field component B_x_ by a factor of 53, and the strongest gradient dB_x_/dz by a factor of 7. To allow the coils to be used in environments with temporally-varying magnetic interference a dynamic nulling system was developed with a shielding factor of 40 dB at 0.01 Hz. Reducing the number of coils required and incorporating dynamic nulling should allow for greater take-up of this technology. Interactions of the coils with the high-permeability walls of the MSR were investigated using a method of images approach. Simulations show a degrading of field uniformity which was broadly consistent with measured values. These effects should be incorporated into future designs.

## Introduction

Magnetoencephalography (MEG) non-invasively measures magnetic fields outside the head generated by neuronal currents in the brain, providing information on brain function with high spatial and temporal resolution^1,2^. The MEG signal is commonly on the level of tens of femtotesla (fT), significantly weaker than the magnetic interference from many other sources^2^. To measure these minute fields, state-of-the-art MEG systems use superconducting quantum interference devices (SQUIDs) and are housed inside a magnetically shielded room (MSR). The SQUIDs generally require cooling to a temperature of 4 K using liquid helium. The need for cryogenic cooling results in a one-size-fits-all sensor arrangement where the subject must remain with their head fixed in position inside the MEG helmet during a scan. This limits the range of subject groups that can be investigated, since, for example, a child’s head will be far from the sensors, resulting in decreased sensitivity to brain activity, and although baby-focused MEG devices do exist, lifetime-compliance cannot be achieved with a single system^3^. The neuroscientific questions that can be addressed are also restricted, as subjects have to remain relatively still during measurements. Implementation of tasks requiring significant subject movement, such as social interaction or spatial navigation is therefore challenging.

As a result of the limitations of SQUID-based measurements of neuromagnetic fields, MEG systems built using optically pumped magnetometers (OPMs) are garnering considerable interest^4–9^. OPMs measure changes in magnetic field by exploiting the optical properties of a heated vapour cell containing spin-polarised alkali atoms^10^. As a result of a considerable effort in miniaturisation^6^, it is now possible to produce OPM sensors which can be flexibly placed on the scalp of a subject. Simulation studies suggest that a four-fold increase in signal strength and improved accuracy in source reconstruction can be achieved through the resulting reduction in source-to-sensor distance relative to typical SQUID-based MEG systems^11,12^. With such a wearable setup the subject is also notionally free to move during an experiment, provided the OPMs remain in a fixed position with respect to the head.

QuSpin OPMs are commercially available magnetic field sensors, which operate in the spin exchange relaxation free regime with a sensitivity of <15 fT/√Hz^13^. In this manuscript we make use of two sensor types: Generation 1 and 2 QuSpin sensors. Generation 1 sensors have a sensitive volume of gas located at ~6 mm from the sensor surface with external dimensions 13 × 19 × 110 mm^3^. Generation 2 sensors have an approximately 6.5 mm distance  from the sensitive volume to outer surface, but with a more compact casing of 12 × 17 × 24 mm^3^ size (https://quspin.com/products-qzfm/). With these sensors, high sensitivity operation can only be achieved for fields measured within a narrow operational range of up to ±5 nT^13^. The passive shielding employed in a typical 2-layer mu-metal MSR, reduces the remnant Earth’s field to a few 10’s of nT. Further reduction of the field in the vapour cell can be achieved by using “on-sensor” coils wrapped around the vapour cell. At the start of an experiment these coils produce a compensation field, locked and optimised for a fixed sensor position and orientation^14^. However, with this approach translation or rotation of the OPM in the spatially varying remnant field inside a MSR can easily produce field changes which exceed the sensor’s operational range, thus rendering the sensor’s output unusable until it returns to its original position.

Our previous work^5,7,8^ showed that the remnant field in a MSR can also be nulled using a set of six, bi-planar coils mounted on large planes of dimensions 1.6 × 1.6 m^2^, separated by 1.5 m, which compensate B_x_, B_y_ and B_z_ as well as the three dominant first-order spatial field gradients G_x_ = dB_x_/dz, G_y_ = dB_y_/dz and G_z_ = dB_z_/dz. These coils were used in a MSR (Vacuumschmelze, Hanau, Germany) comprised of two layers of mu-metal and one layer of aluminium to reduce the dominant static field component, B_x_, by a factor of 46 from 21.8 ± 0.2 nT to 0.47 ± 0.08 nT, and the dominant gradient, G_x_, by a factor of 13 from 7.4 nT/m to 0.55 nT/m^7^. The integration of these coils with a scalp-mounted OPM array allowed high quality MEG data to be recorded whilst the subject made large (±10 cm and ±35°) movements^7^. With this set-up, MEG recordings have been made while unconstrained subjects perform naturalistic experiments, such as a ‘bat and ball’ game^5^, as well as when performing a language lateralisation task^8^.

The bi-planar coils thus form a crucial element of an OPM-MEG scanner that allows measurements to be made on a moving subject, but their construction is time-consuming due to the large size of the coils and the significant number of coil layers that are required. The distribution of the weight of the current carrying wires and structural rigidity of the coil-set also have to be taken into account during coil construction.

**Figure 1:**
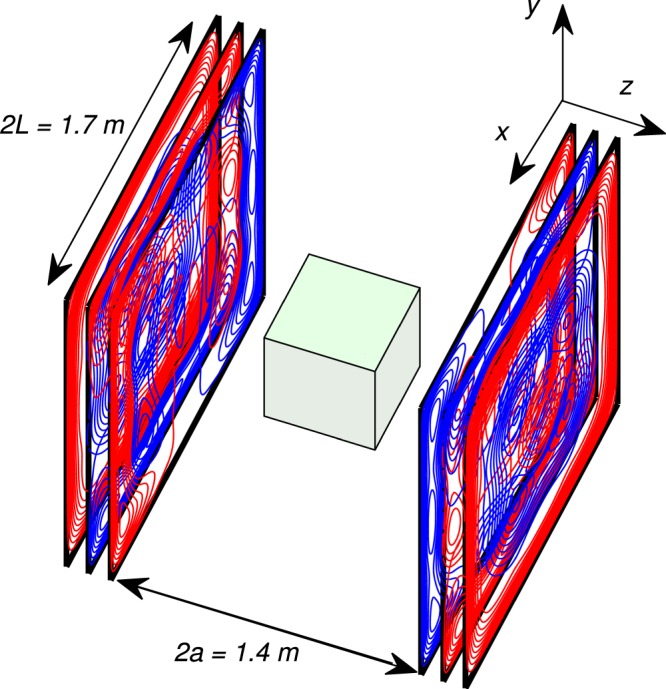
Geometry of the coil system. Two square planes of side length 2 L = 1.7 m placed a distance 2a = 1.4 m apart. Each coil pair is simultaneously designed to produce a homogeneous field and a homogeneous  field gradient over a region at the centre of the coils. The three coil layers on each plane are spaced by ~1 mm in the constructed system.

Here, we show that by exploiting the natural symmetry shared by pairs of coils (B_x_ and G_x_, B_y_ and G_y_ and B_z_ and G_z_) the number of coils that need to be constructed to produce a simple field-nulling system can be reduced from six to three, as illustrated in Fig. 1. We describe the harmonic-minimisation approach that allows optimisation of planar current distributions to produce both homogenous fields and spatial field gradients. The performance of coils generated using the joint design method is compared with that of coils designed to produce only a uniform field or field gradient. We also detail a proportional integral derivative (PID) control method, which nulls the static field and field gradients automatically by independently optimising the currents in each plane based on reference measurements. The static field compensation achieved using this method is quantified by comparing field maps measured in a single plane in the MSR before and after field compensation.

The previous six-coil system provided only static field compensation with coil currents locked during an experimental session. As well as the large static components, significant temporal variation of the remnant magnetic field by as much as 10’s of nT over the period of a single experiment (10–20 minutes) can occur depending on the environment. This drifting background field level has a detrimental effect on the open-loop OPM sensor gain. This gain varies by more than 10% over the full dynamic range of ±5 nT^13^. For accurate MEG measurements it is desirable that the sensor gain varies as little as possible, and as a result dynamic compensation of the background magnetic field is also required for sensor operation. Iivanainen and colleagues have described a field-nulling system which allows the operation of a QuSpin OPM array inside a poorly shielded environment by continually updating currents in a series of coils to account for dynamic variations in the remnant field^15^. In their work, dynamic nulling prevented significant sensor gain changes over the course of an experiment and reduced the peak-to-peak drift of the field from 1.3 to 0.4 nT^15^.

The three coils here were designed for use at a city-centre site located directly above a London Underground railway line. This environment produces unpredictable, temporally varying magnetic fields large enough to send OPMs outside of their operational range and frequent enough to make even a 10-minute scan unfeasible. To correct for this, a dynamic PID-based feedback controller was developed to continually update coil currents during a session in order to null reference measurements of B_x_, B_y_ and B_z_. Empty room recordings with and without the dynamic nulling were compared whilst noting the number of trains which passed beneath the lab during these recordings.

Discrepancies between the predicted and measured coil performance may be caused by interactions between the coils and the high-permeability mu-metal walls of the MSR. This was not considered in the process of coil design here, though similar interactions have been incorporated into gradient coil design methods for MRI^16^. It has been shown that this interaction can be modelled by a method of images approach^17,18^. This was utilised to simulate the effects of interactions on the efficiency and homogeneity of the designed coils when operated inside a MSR. Results are compared to the measured performance and the impact this may have on future coil designs is discussed.

## Coil Design Theory

**Figure 2:**
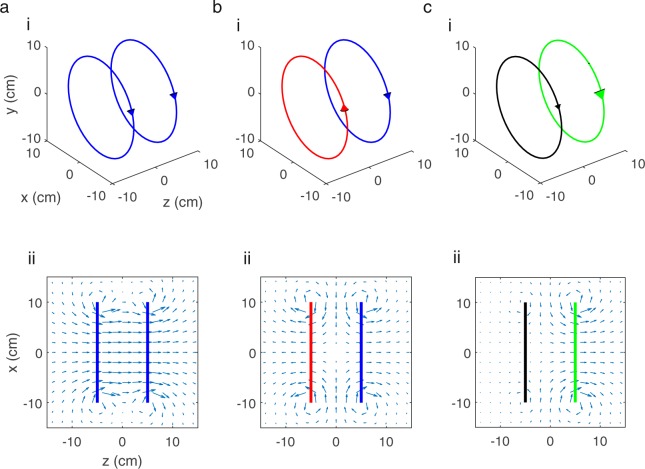
The field produced by a pair of co-axial loops varies with the strength and direction of the applied currents. (**a**) (i) The Helmholtz coil consists of two current-carrying loops. The same current is applied to each loop making the stream function symmetric in x, y and z. (ii) A homogeneous field B_z_ is produced through the centre of the loops. (**b**) (i) The current applied to one of the loops is reversed (now making the stream function anti-symmetric in z) (ii) In this case a B_z_ field which linearly varies with z position (constant dB_z_/dz) is produced. (**c**) (i) Independently driving the two loops. (ii) A desired field and gradient can be simultaneously produced.

We can characterise a current distribution ***J*** that is confined to an x-y plane using a two-dimensional stream function S(x, y), chosen such that $\nabla S\times \hat{{\boldsymbol{z}}}={\boldsymbol{J}}$ (since ∇.***J*** = 0). The wire paths of a coil design that represents the current distribution correspond to the contours of the stream function. Planar current distributions which produce a uniform field along one of the Cartesian axes have shared symmetry with current distributions that generate specific field gradients, and this sharing of symmetry also applies to the stream functions. For example, in the case of a Helmholtz coil, two co-axial loops of radius a carrying the same current and placed a distance a apart in z will produce a uniform field B_z_ along the z direction as shown in Fig. 2a. In this instance the stream function required to produce the field is symmetric in x and y, and also symmetric in z (as the loops carry the same current). If the current in one of the loops is reversed with respect to the other (so the stream function is now anti-symmetric in z, and symmetric in x and y) then the result is a gradient in B_z_ along the z direction (i.e. a dB_z_/dz coil) as shown in Fig. 2b. If the two loops are driven independently the currents can be set to produce both a field, and a field gradient simultaneously as shown in Fig. 2c. This involves applying the sum of the currents needed to produce a field and a field gradient to one loop and the difference to the other loop.

Considering the symmetry of the coil stream functions required to generate each of the three uniform fields, the x-y symmetry is the same for B_x_ (anti-symmetric in x, symmetric in y and anti-symmetric in z) and G_x_ (anti-symmetric in x, symmetric in y and symmetric in z) coils, B_y_ (symmetric in x, anti-symmetric in y and anti-symmetric in z) and G_y_ (symmetric in x, anti-symmetric in y and symmetric in z) coils, and B_z_ (symmetric in x, symmetric in y and symmetric in z) and G_z_ (symmetric in x, symmetric in y and anti-symmetric in z) coils. Through a combined design process and careful control of the applied currents, three coil pairs can therefore be used simultaneously to generate the uniform fields and the associated gradients.

Here, we used a harmonic minimisation approach based on previously described expressions for planar magnetic field coils^19–21^ to design the coil pairs. The two-dimensional Fourier transform of the magnetic field $\tilde{{\boldsymbol{B}}}({k}_{x},{k}_{y},z)$ over the x-y plane at a position z in the region −a < z < a between the two planes can be written as:

1 $$\tilde{{\boldsymbol{B}}}({k}_{x},{k}_{y},z)={\mu }_{0}\{[i{k}_{x}\hat{{\boldsymbol{x}}}+i{k}_{y}\hat{{\boldsymbol{y}}}]\begin{array}{c}\sinh \\ \cosh \end{array}({k}_{r}z)-{k}_{r}\hat{{\boldsymbol{z}}}\begin{array}{c}\cosh \\ \sinh \end{array}({k}_{r}z)\}\tilde{S}({k}_{x},{k}_{y}){e}^{-{k}_{r}a},$$

where *k*_*r*_ = *(k*_*x*_^2^ + *k*_*y*_^2^)^*1/2*^ and $\tilde{S}({k}_{x},{k}_{y})$ is the two-dimensional Fourier transform of the stream function. The upper/lower terms define the *z*-symmetry where the stream function has the same (symmetric in z)/opposite (anti-symmetric in z) sign on each plane.

The stream function is then parameterised as a two-dimensional Fourier series confined to the region $|x|,|y| < L$ ($z=\pm a$) on the two coil planes, so that^20^:

2 $$S(x,y)=\mathop{\sum }\limits_{n=1}^{N}[{\alpha }_{n}\,\cos (\frac{\pi }{2}(2n-1)\frac{x}{L})+{\beta }_{n}\,\sin (\frac{\pi nx}{L})]\times \mathop{\sum }\limits_{m=1}^{M}[{\gamma }_{m}\,\cos (\frac{\pi }{2}(2m-1)\frac{y}{L})+{\delta }_{m}\,\sin (\frac{\pi my}{L})],$$

where coefficients *α*_*n*_, *β*_*n*_, *γ*_*m*_ and *δ*_*m*_ are used to weight the different harmonics in the series. By applying symmetry conditions to the stream function, a sub-set of the harmonic combinations in Eq. (2) can be used to design each coil. For example, in the case of a balanced B_x_ and G_x_ coil, the stream function is required to be anti-symmetric in x and symmetric in y. These constraints allow the stream function to be written as

3 $${S}_{({B}_{x}{G}_{x})}=\mathop{\sum }\limits_{n=1}^{N}\,\mathop{\sum }\limits_{m=1}^{M}\,[{\lambda }_{nm}\,\sin (\frac{\pi nx}{L})\cos (\frac{\pi }{2}(2m-1)\frac{y}{L})].$$

These stream function equations are written for ease of notation in the form $\,S(x,y)=\mathop{\sum }\limits_{j=1}^{N\times M}{\lambda }_{j}{S}_{j}(x,y)$ with *j* = *(n−1)N* + *m*. Continuing with the case of the balanced B_x_/G_x_ coil, the contribution to the field B_x_ in the x-direction, *b*_*xj*_(***r***_***i***_), from the *j*^*th*^ component of the stream function can be expressed (defining the z-symmetry to be antisymmetric, i.e. we choose cosh in Eq. (1)) using Eq. (1) as

4 $$\,{b}_{xj}({{\boldsymbol{r}}}_{{\boldsymbol{i}}})=twoDFT{(i{\mu }_{0}{k}_{x}{\tilde{S}}_{j}({k}_{x},{k}_{y}){e}^{-{k}_{r}a}\cosh ({k}_{r}{z}_{i}))|}_{{x}_{i},{y}_{i}},$$

where twoDFT denotes the two-dimensional Fourier transform. The contribution to the field from the gradient component G_x_ in the x-direction, *g*_*xj*_(***r***_***i***_), from the *j*^*th*^ component of the stream function can also be expressed (defining the z-symmetry to be symmetric, i.e. we choose sinh in Eq. (1)) using Eq. (1) as

5 $$\,{g}_{xj}({{\boldsymbol{r}}}_{{\boldsymbol{i}}})=twoDFT{(i{\mu }_{0}{k}_{x}{\tilde{S}}_{j}({k}_{x},{k}_{y}){e}^{-{k}_{r}a}\sinh ({k}_{r}{z}_{i}))|}_{{x}_{i},{y}_{i}}.$$

Defining ${\tilde{S}}_{j}$ in terms of the reduced variables $\,x^{\prime} =x/L$, $y^{\prime} =y/L$ (${k}_{x}^{^{\prime} }={k}_{x}L$, $\,{k}_{y}^{^{\prime} }={k}_{y}L$) allows expression of the Fourier transform of Eq. (3) as

6 $${\tilde{S}^{\prime} }_{j}({k^{\prime} }_{x},{k^{\prime} }_{y})\propto [{\rm{sinc}}((n-1/2)\pi -{k^{\prime} }_{y})+{\rm{sinc}}((n-1/2)\pi +{k^{\prime} }_{y})]\times [{\rm{sinc}}(m\pi -{k^{\prime} }_{x})-{\rm{sinc}}(m\pi +{k^{\prime} }_{x})].$$

This can be substituted into Eqs (4) and (5) to find the field at position ${{\boldsymbol{r}}}_{{\boldsymbol{i}}}$ due to each component of the stream function. Similar calculations can be performed by imposing the symmetry conditions needed for the other coils.

*λ*-coefficients are then chosen to minimise a functional containing three parts^20^: the spatial deviation of the field term (B_x_) over an array of target points, the spatial deviation of the field gradient term (G_x_) and the power dissipated in the coil. The functional is written as:

7 $$F=\mathop{\sum }\limits_{i=1}^{I}{|{B}_{t}({{\boldsymbol{r}}}_{{\boldsymbol{i}}})-{b}_{x}({{\boldsymbol{r}}}_{{\boldsymbol{i}}})|}^{2}+\alpha \mathop{\sum }\limits_{i=1}^{I}{|{g}_{t}{z}_{i}-{g}_{x}({{\boldsymbol{r}}}_{{\boldsymbol{i}}})|}^{2}+\omega P.$$

Here, from the B_x_ term *B*_*t*_(***r***_***i***_) is the desired (or target) field at position ***r***_***i***_ and ${b}_{x}({{\boldsymbol{r}}}_{{\boldsymbol{i}}})=\sum _{j}{\lambda }_{j}{b}_{xj}({{\boldsymbol{r}}}_{{\boldsymbol{i}}})$ is the calculated field at ***r***_***i***_. For the G_x_ term *g*_*t*_*z*_*i*_ is the desired (or target) field chosen to vary linearly with position *z*_***i***_ and ${g}_{x}({{\boldsymbol{r}}}_{{\boldsymbol{i}}})=\sum _{j}{\lambda }_{j}{g}_{xj}({{\boldsymbol{r}}}_{{\boldsymbol{i}}})$ is the calculated field at ***r***_***i***_. The set of position vectors ***r***_*i*_ _= 1 *to I*_ define target points within the volume at which a homogeneous field or field gradient is required. The linear weighting *α* applied to the field gradient minimisation can be altered to balance the strength of the field and gradient terms. *P* is a tuneable power dissipation term which can be upweighted by increasing the weighting coefficient *ω *to reduce the complexity of the designed coils^20^.

Here the functional is minimised by choosing the weights *λ*_*j*_ which satisfy *dF*/d*λ*_*j*_ = 0. The set of derivatives can be cast as a set of linear simultaneous equations in matrix form, whose solution is found by identifying the pseudo-inverse matrix^7^. The wire paths of the coils are then extracted as contours of the optimised stream function.

## Methods

### Coil design and construction

MATLAB (The MathWorks Inc.) was used to produce designs for three coils allowing simultaneous compensation of B_x_ and G_x_, B_y_ and G_y_ and B_z_ and G_z_. The dimensions of the system were limited by the size and layout of the MSR which also contains a SQUID-based MEG system (CTF, Coquitlam, BC, Canada). Final bi-planar dimensions were chosen to be a = 0.7 m and L = 0.85 m as shown in Fig. 1. The number of harmonics, power term weighting and field/gradient term weightings were chosen manually to produce homogeneous fields and field gradients (to within ± 5%) over a central volume of 40 × 40 × 40 cm^3^. The magnetic field for each coil was evaluated over a 2.5-cm-resolution, 3D grid using the elemental Biot-Savart law. A numerical value of the field, or field gradient homogeneity, *H* was computed by assigning a value of 1 to every element in the 3D grid where the homogeneity condition was met, and a 0 where it was not. The homogeneity is then reported as a percentage of the number of 1’s versus the total number of grid points. The balanced B_x_/G_x_ and B_y_/G_y_, coils were designed using 36 harmonics with N = M = 6, and the field was evaluated over I = 320 target points. The balanced B_z_/G_z_ coil was designed with 36 harmonics with N = M = 6 and the field was evaluated over I = 256 target points. Note that fewer target points are required to limit the field variation in the B_z_/G_z_ coil due to the high degree of rotational symmetry of these coils about the z-axis.

Contours of the optimised stream function were connected to form continuous wire paths, as shown in Supplementary Information. The coils were mounted on two sheets of medium-density fibreboard (MDF) measuring 1.8 × 1.8 m^2^ with legs which place the centre of the coils 1.1 m from floor level. Coil designs were printed and then hand-traced onto thin paper sheets, these sheets were attached to the board with wallpaper paste. Copper wire (enamelled, diameter of 0.56 mm) was laid on each path and fixed in place using masking tape. The total thickness of each coil layer is around 1 mm. The three coils were layered by repeating these steps.

Each planar coil unit was connected to a coil driver (4 V, low-noise, six drivers in total, one per coil unit), which was controlled using a 16-bit NI-9264 DAC voltage output module interfaced with LabVIEW (National Instruments (NI) Corporation, Austin, TX). Resistors were added in series to each coil unit so that fields of around 40 nT or field gradients of around 25 nT/m could be produced from a 4 V output.

Prior to their use in balanced mode, the coils were connected in standard, bi-planar arrangements (with equal or equal and opposite current in each plane^7^) to test their performance when producing each of B_x_ and G_x_, B_y_ and G_y_ and B_z_ and G_z_. The coil efficiencies (field or field gradient per unit current) were measured by using an array of three Gen-1 QuSpin OPMs operating in a mode where the field is measured only along the sensor’s radial axis. These OPMs were then oriented so as to measure the field component of interest.

A linearly increasing current was applied to each bi-planar coil and the change in field measured as a function of this applied current. For B_x_/G_x_ and B_y_/G_y_ the OPMs were positioned approximately at the centre of the bi-planar coils and spatially separated by 8 cm in the z direction (such that the field was measured at roughly z = −8 cm, 0 cm and +8 cm at x = 0 cm, y = 0 cm). For the B_z_/G_z_ measurements, the long axes of the sensors were lined up in the measurement plane, which meant that the field had to be sampled at locations with a greater spatial separation in the z direction (field measured at z = −17 cm, z = 0 cm and z = + 17 cm at x = 0 cm, y = 0 cm). For comparison, the fields and gradients produced by the bi-planar coils were also calculated using the elemental Biot-Savart law. These measurements were carried out in the MSR at the University of Nottingham (Vacuumschmelze, Hanau, Germany) comprised of two layers of mu-metal and one layer of aluminium.

### Comparing design methods

To compare the performance of the balanced coils designed using the combined method to that of coils designed just to produce a uniform field or field gradient, three coils were investigated: a coil designed to produce B_x_; a coil designed to produce G_x_; a coil designed to produce B_x_ and G_x_. For each case the number and location of target points and the weighting of the power dissipation term were kept constant. The dimensions of the coils were a = 0.7 m and L = 0.85 m as above. The field and gradient produced by these coils in a bi-planar arrangement was calculated using the elemental Biot-Savart law. Fields were then normalised to their values at the centre to allow evaluation of the deviation of the field and gradient over a 40 × 40 × 40 cm^3^ volume (evaluated over a 2.5 cm resolution 3D grid). The homogeneity *H* is also reported for each case, using the measure described in section 3.1. As before, less than ±5% deviation over the full volume is desired.

### Static field nulling

Although in their standard measurement mode QuSpin OPMs have an operational range of ± 5 nT they can also be operated in a “field-zeroing” mode^13,14^. This mode produces the zero-field environment which is required for sensitive measurements based on changes in transparency of the vapour cell around a zero-field resonance^22^. Each OPM contains a set of three orthogonal “on-sensor” coils sited around the vapour cell. In field-zeroing mode currents are applied to the on-sensor coils under feedback control to maximise the optically-measured zero-field resonance. Once optimised currents have been found, the amplitude of the static magnetic fields in two directions perpendicular to the laser beam can be inferred from the applied current and the known field per unit current of each on-sensor coil. This field is reported in the range 1 to 50,000 pT.

**Figure 3:**
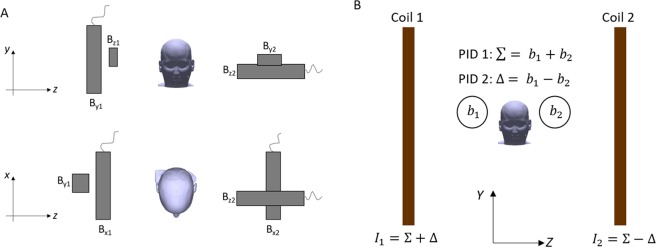
Automated field nulling based on reference measurements. (**A**) A reference array of four Gen-1 QuSpin OPMs is used to measure the vector field components B_x_, B_y_ and B_z_ at two locations separated in the z direction. (**B**) To drive a single coil two PID controllers are used. One uses Σ to drive the sum of the field at the two locations (driving for example the B_x_ field down towards zero). The second PID uses ∆ to drive the difference of the field at the two locations (the gradient G_x_) towards zero. Currents which are the sum and difference of these values are then applied to the planes.

Using the field-zeroing process four Gen-1 QuSpin OPMs were used to measure the three vector field components, at two positions spatially separated in the z-direction by ~40 cm, as shown in Fig. 3A. The measurements from the sensors are combined to form estimates of the magnitudes of B_x_, B_y_ and B_z_ and of G_x_, G_y_ and G_z_ (for example, G_x_ is found through subtraction of two measurements of B_x_).

To automatically null the field over the reference array, two proportional integral derivative (PID) controllers are driven in tandem and used to identify the optimal currents to pass through the coil windings in each plane. Taking the example of nulling the B_x_ and G_x_ fields_,_ the reference array produces two measures of B_x_ (b_x1_ and b_x2_) at locations which are spatially separated in *z*. The first controller varies a parameter ∆ in order to reduce b_x1_ − b_x2_, which provides a measure of the field gradient G_x_. The second controller varies a parameter ∑ in order to reduce b_x1_ + b_x2_, which provides a measure of B_x_. Currents I_1_ and I_2_ are then supplied to the two different coil planes, where I_1_ = ∑ + ∆ and I_2_ = ∑−∆. The balancing of the terms results in simultaneous reduction of the field and field gradient towards zero. A LabVIEW-based controller was developed to implement this algorithm by linking to the reference array and coil drivers. Once nulling is completed the PIDs are switched off, and stable currents are applied to the coils. This process is outlined in Fig. 3B.

### Field mapping

To quantify the reduction in field and field gradient, field maps were taken over a single plane before and after the coils were used for field nulling. A 40 × 40 cm^2^ region in the x-z plane lying between the reference sensors (which were separated by 40 cm in z) was mapped. By taking two measurements using the field-zeroing mode of a single Gen-1 QuSpin OPM at each position on a 10-cm grid (changing the orientation of the sensor to allow the field to be measured in three dimensions) over the plane, a representation of the static field can be obtained. The data were linearly interpolated (using the MATLAB interp2 function) onto a 1-cm resolution grid for visualisation, and field maps produced to show changes in magnitude and spatial variation.

The average field is calculated as the mean over each point in the interpolated grid with the associated standard deviation given as the error. A measure of the gradient is found from the difference in field (∆B) divided by the difference in z position (∆z). The difference of the field values measured at z = −20 cm and z =  + 20 cm were taken so that ∆z = 40 cm. These measurements were carried out in the MSR at the University of Nottingham.

### Dynamic field nulling

The balanced bi-planar coils described here were intended for use in an OPM-MEG system to be operated inside a MSR (Vacuumschmelze, Hanau, Germany) comprised of two layers of mu-metal and one layer of aluminium, which is sited in the basement of the Wellcome Centre for Human Neuroimaging at University College London (UCL). This city centre site is close to the Piccadilly Line of the London Underground as it runs between Russell Square and Holborn underground stations at a depth of around 33 m (maps generated from Transport for London data available here - https://www.dansilva.co.uk/down-underground). Given the MSR is in the basement of the Wellcome centre, and using Citymapper (www.citymapper.com) to estimate the distance to the line at ground level (around 85 m), we estimate the railway line to be ~90 m away from the MSR. As a consequence of proximity to the passing underground trains the background magnetic field in the MSR varies by several nT over a 10-minute period. This drift pushes the OPMs close to the edge of their dynamic range and causes gain changes. In order to address this issue, we developed a dynamic PID-based set-up that could be used to control the currents in the balanced, bi-planar coils to keep the ambient field within the sensors’ operational range during recordings, minimising any impact on their performance.

An array of 15, Gen-2 QuSpin OPMs were placed in an empty 3D printed scanner-cast^11^. Three of these sensors, which were placed in slots oriented in the x, y and z directions so that they measured the field components B_x_, B_y_ and B_z_ along their radial axes, were selected as reference sensors. The sensors were operated in the single-axis measurement mode with the dynamic range set to ±5 nT and data were recorded using a National Instruments 16-bit NI-9205 ADC interfaced with a LabVIEW controller. The voltage outputs from LabVIEW for driving the coils were controlled using the NI-9264 DAC. The controller sampled data from all 15 sensors at a 6,000 Hz sampling rate and the data was saved in chunks of 50 samples. For each of these 50-sample chunks the data from the three reference sensors was selected and passed to a digital, first-order, low-pass Butterworth filter with a 1 Hz cut-off frequency (chosen such that the controller was addressing only the low frequency fluctuations in the remnant magnetic field). The average of these 50 samples was then taken.

The averaged values were then fed into three PID controllers (one for controlling each of the B_x_, B_y_ and B_z_ fields). These controllers were manually tuned by observing the system response to variation in the proportional and integral gains (derivative gains were set to zero) to provide stable nulling without interfering with each other, as cross-talk can be introduced between the coils and reference sensors. For example, the effect of poor coil or reference sensor positioning could lead to a measurable effect of the B_x_ coil on the B_y_ reference sensor, in turn leading to an over-correction on the B_y_ coil which impacts on the B_x_ reference sensor and so on. A well-tuned loop can readily compensate for these ‘cross-talk’ effects. The PID output voltages were applied to the coils on the two planes such that uniform field components were produced (i.e. voltages of the same sign were applied to the coils for generating B_z_, and of opposite sign for producing B_x_ and B_y_). Data from the remaining 12 sensors were also recorded. During each recording, data was taken from the Transport for London website for trains which ran on either of two routes: eastbound from Holborn to Russell Square and westbound from Russell Square to Holborn. Whilst this does not provide the exact time that trains came in closest proximity to the MSR, it does indicate the number of trains that crossed beneath the lab during the recordings.

A recording with the nulling off was taken between 17:22 and 17:32, while a recording with nulling- on was made between 17:35 and 17:45. These times fall during the ‘rush hour’, during which there is heavy train activity (trains arrive roughly every 2 minutes on the Piccadilly line around this time), thus providing a challenging environment for the measurements.

### Investigating interactions with the shielded room

The fields that are produced by the coils may interact with the walls of the MSR and result in deviations from the predicted performance. At a boundary between materials of different relative magnetic permeability μ_r_, the following conditions apply: to obey Ampere’s law the tangential component of the magnetic field **H** must be continuous across the surface, and to obey Gauss’s law the normal component of **B** must also be continuous^23^. Since **B** takes the form **B** = μ_0_μ_r_**H** inside the material and μ_r_ can be >80,000 for mu-metal, while μ_r_ ~ 1 in air, the field must abruptly change direction at an interface between air and mu-metal to satisfy the two boundary conditions^23^, and in particular on the air-side of the boundary the tangential components of the **B** and **H** fields must be approximately zero. Under these circumstances the field variation within the MSR can be evaluated by addition of a set of virtual mirror currents produced by simple reflection of current-carrying elements of the coils at each of wall of the room. For magnetostatics’ problems, this “method of images” approach is not very sensitive to the exact value of μ_r_, just requiring that μ_r_» 1 since deviations from the field variation it predicts scale as (μ_r_)^−1^ which is negligible for mu-metal^24,25^. To ensure that the boundary conditions are properly fulfilled, reflections have to be calculated recursively so that the mirrored currents are themselves mirrored. Here, the effect that the mu-metal walls have on the form of the field produced by the balanced coils operating inside the MSR has been modelled via the addition of a truncated set of mirror currents^17,18^.

Considering a single current element, reflections are made at each of the internal faces of the shielded room and the direction *d****l*** reversed according to ${d}{\boldsymbol{l}}={d}{\boldsymbol{l}}-2({d}{\boldsymbol{l}}.\hat{{\boldsymbol{n}}})$ where $\hat{{\boldsymbol{n}}}$ is the unit vector perpendicular to the plane in which the element was reflected. After consideration of each internal face of the MSR, this results in the production of 6 mirrored current elements. Each of these mirrored elements is then also mirrored by reflection at the five walls of the MSR that were not involved in the original reflection. This process should be repeated ad infinitum fully to satisfy the boundary conditions on the magnetic fields at the MSR’s internal walls. Accounting for redundancies there are 6 first order mirror elements, 18 second order mirrors, 38 third order mirrors and so on. The additional field produced by each of the mirrored elements is calculated using the elemental Biot-Savart law. Here we only considered reflections up to 3^rd^ order. This order was chosen as the change in coil efficiency (at the centre of the planes) with reflections up to second order was found to be around 30%, while the additional contribution from third order mirrors only modified the efficiency by a further ~5%.

We conducted a simple experiment to test the mirroring method in the UCL MSR, which has internal dimensions of 3.85 × 2.4 × 3 m^3^. A single coil plane was placed parallel, and as close as possible, to one of the 3.85 × 2.4 m^2^ walls of the MSR. The coil plane was offset from the centre of the MSR in by 0.6 m and −0.2 m in x and y and due to the legs of the wooden support structure the centre of the coil was displaced by 25 cm in the *z*-direction from the proximal wall of the MSR. A 0.1 Hz sinusoidally oscillating voltage with amplitude of 1 V (equivalent to 8.1 mA current) was applied to the B_z_/G_z_ coil on this plane and the z-component of the magnetic field was measured at a position 5 cm displaced from the centre of the coil in the z-direction away from the proximal wall of the MSR (but centred in x and y) using a single-axis fluxgate magnetometer (Fluxmaster, Stefan Mayer Instruments, Dinslaken, Germany). The coil plane and fluxgate magnetometer were then further displaced from the wall in steps of 20 cm in the z-direction until the setup could not be moved any further due to the presence of a cryogenic MEG system (CTF, Coquitlam, BC, Canada). A final displacement of 85 cm from the wall was achieved. At each position the fluxgate magnetometer was similarly positioned relative to the coil. For comparison the z-component of the field was calculated, with, and without the effect of mirroring by applying the elemental Biot-Savart law to the B_z_/G_z_ coil and its reflections.

The effects of the interactions on the bi-planar coils was then investigated by simulation. The field was calculated over a 40 × 40 × 40 cm^3^, 2.5 cm resolution 3D grid at the centre of the two coil planes and the results compared to measurements of the coil field or field gradient per unit current. Three cases were studied: first, when no mirrors were considered, second when the coils were positioned centrally in the MSR at the University of Nottingham and mirrors up to third order were included, and finally when the coils were positioned as they are during an experimental session and mirrors up to third order were considered. For each case a box plot of the full range of fields calculated at all grid positions was produced to show the effects of the mirror currents on both the homogeneity and the mean level of the produced field.

The effects were investigated for the three coil pairs when producing B_x_, B_y_ and B_z_. The MSR considered here has internal dimensions of 3.85 × 2.4 × 3 m^3^ and during an experiment the centre of the bi-planar coil set is displaced from the centre of the room by + 0.2 m in y and −0.25 m in x (with zero offset in z).

## Results

### Coil designs

**Figure 4:**
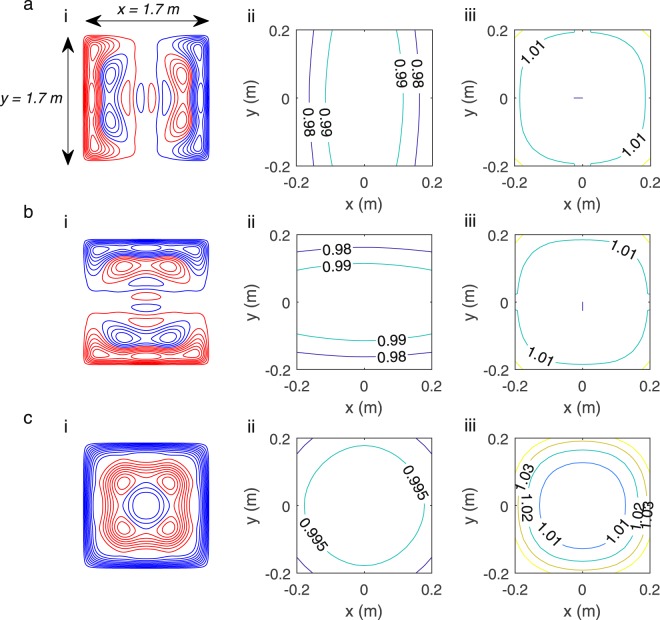
Final coil designs and simulated fields. Wire paths (i) and field (ii) or field gradient contours (iii) for the: (**a**) B_x_ and G_x_ coil, (**b**) B_y_ and G_y_ coil and (**c**) B_z_ and G_z_ coil, respectively. Red and blue denote wires with opposite senses of current flow. The contours of the field or field gradient in the plane at z = 0 m, for |x|, |y| < 0.2 m (z = 0.025 m, for |x|, |y| < 0.2 m for G_z_) are shown. The field values are normalised to the field at the centre of the plane or a value slightly off-centre when the central field is zero.

Figure 4 shows wire paths (i) for the (a) B_x_ and G_x_, (b) B_y_ and G_y_ and (c) B_z_ and G_z_ coils respectively. The elemental Biot-Savart law was applied to the wire-paths to evaluate the spatial variation of the field (ii) or field gradient (iii) produced, when the coils are wound in a bi-planar arrangement. The field was calculated over a 3D array of points covering a region of 40 × 40 × 40 cm^3^. Contours are shown over a plane at z = 0 m; |x|, |y| < 0.2 m for the case of B_x_, B_y_, B_z_, G_x_, and G_y_ and over the plane at z = 0.025 m; |x|, |y| < 0.2 m for the G_z_ case. The fields were normalised to the value at the centre of the two planes (or a value positioned slightly off-centre when the field is zero at the centre). The homogeneity of all the produced fields was *H* = 100%.

Table 1 shows calculated and measured values for the bi-planar coil efficiencies and single plane inductances. The errors quoted for the field generating coils are the standard deviation from the mean measured across three OPMs spatially separated in z, while the errors in the gradient generating coils are the differences in the two gradient values calculated from the three OPM measurements. The inductances were calculated from the stream functions using previously published expressions^26^.

**Table 1:** Comparing theoretical and measured values (in brackets) of the efficiency for the coil designs when wound as field and gradient coils.

Coil	Efficiency as field coil (nT/mA)	Efficiency as gradient coil (nT/m/mA)	Single plane Inductance (μH)
B_x_ and G_x_	0.74 (0.57 ± 0.01)	1.20 (1.11 ± 0.02)	169 (192)
B_y_ and G_y_	0.74 (0.47 ± 0.02)	1.20 (0.98 ± 0.02)	169 (193)
B_z_ and G_z_	3.9 (3.6 ± 0.3)	4.6 (4.5 ± 0.7)	379 (385)

Errors are the standard deviation from the mean of measurements taken across three OPMs spatially separated in z. The single plane inductances are also reported.

### Comparing design methods

**Figure 5:**
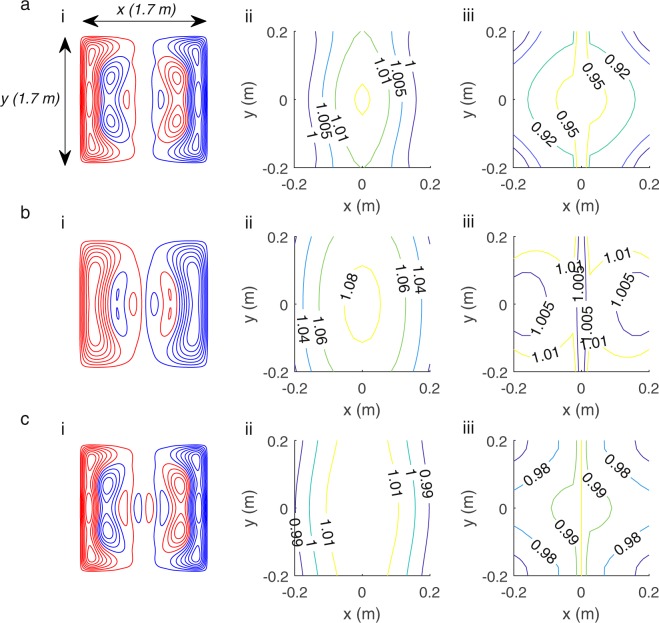
Investigating the performance of the joint design method versus only producing a single field component. (**a**–**c**) show for coils optimised to produce B_x_, G_x_ and both B_x_/G_x_ respectively: (i) coil wire paths (red and blue denote wires with opposite senses of current flow); (ii) contours of the variation of B_x_ produced by these coils; (iii) contours of the variation of G_x_ produced by these coils. Contours are shown for the plane z = 0.20 m, for |x|, |y| < 0.20 m. The field values are normalised to the field at the centre of the plane or a value slightly off-centre when the central field is zero.

Figure 5 shows for coils optimised to produce (a) B_x_, (b) G_x_ and (c) and both B_x_ and G_x_ (i) designs for coils, (ii) contours of B_x_ and (iii) contours of G_x_. The field and gradient variation is calculated using the elemental Biot-Savart law and is plotted over a plane at z = 0.20 m; |x|, |y| < 0.20 m. This plane is deliberately chosen to be off-centre to highlight the decrease in homogeneity with distance from the centre of the region between the two coil planes.

Comparing the homogeneity of the non-optimised field (e.g. the G_x_ variation produced by the B_x_ coil) shows the combined method improves homogeneity, but with an increase in design complexity. The B_x_ coil had a G_x_ homogeneity of *H* = 76% and the G_x_ coil had B_x_ homogeneity of *H* = 78% compared to *H* = 100% for the optimised field components. The largest deviations of the field and gradient at z = 0.20 m in Fig. 5a(ii-iii) are +2% and −13% respectively. The field and gradient in Fig. 5b(ii-iii) deviate by −9% and +2% respectively. The field and gradient in Fig. 5c(ii-iii) deviate by +3% and −4% respectively. Only the combined design satisfies the condition that both the field and field gradient should be homogenous to within ±5% over a central 40 × 40 × 40 cm^3^ cubic region.

### Field mapping

**Figure 6:**
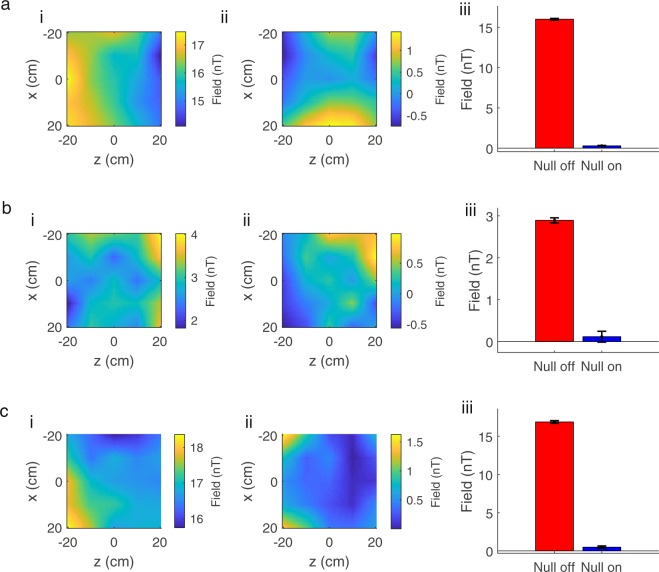
Quantifying the coil performance by measuring the field in a single plane. (**a**–**c**) show for B_x_, B_y_ and B_z_ respectively: (i) a map of the field before nulling is applied; (ii) a map of the field after nulling is applied; iii) the average field magnitude with error-bars showing the spatial standard deviation of measurement before (red) and after (blue) nulling is applied.

Figure 6 shows maps of the spatial field variation in (a) B_x_, (b) B_y_ and (c) B_z_ inside the MSR before (i) and after (ii) the automated nulling process was applied, along with a bar chart (iii) showing the reduction in average field strength. The mean magnitude of the field vector was reduced by a factor of 26 from 23 nT to 0.9 nT. The reduction in the measured individual field components with associated standard deviation from the mean is shown in Table 2. The maps of the field variation are not as smooth as are expected to be found in a source-free region, as a result of inaccuracies in manually setting the sensor position and orientation, and the limited accuracy of measurement that can be achieved when operating the QuSpin sensors in field-zeroing mode.

**Table 2:** Comparing the measured magnitudes of each of the field components before and after field nulling.

Field Component	Nulling OFF	Nulling ON	Reduction Factor
B_x_	16.0 ± 0.1 nT	0.30 ± 0.04 nT	53
B_y_	2.9 ± 0.1 nT	0.11 ± 0.13 nT	26
B_z_	17.0 ± 0.2 nT	0.49 ± 0.15 nT	35
G_x_	6.1 ± 0.9 nT/m	0.9 ± 0.4 nT/m	7
G_y_	2.5 ± 0.9 nT/m	1.8 ± 0.9 nT/m	1.4
G_z_	3.4 ± 0.8 nT/m	1.3 ± 0.3 nT/m	3

Errors are standard deviation from the mean.

### Dynamic field nulling

**Figure 7:**
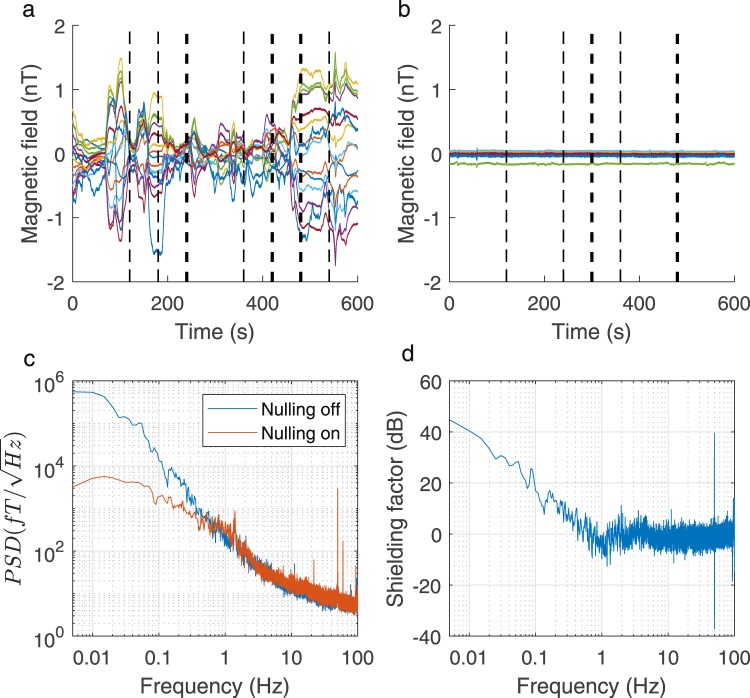
Assessing the performance of dynamic nulling. (**a**) The time-series data from a series of 15 OPMs placed in an empty scanner-cast in an empty MSR with no dynamic nulling applied. Here and in (**b**), vertical dashed lines show the approximate times that a train arrived at either Russell Square or Holborn stations, thick lines indicate two trains arriving at approximately the same time. (**b**) Time-series data from the same OPMs with the dynamic nulling switched on. (**c**) The median power spectral density of the sensor outputs for the two cases across all sensors. (**d**) The shielding factor of the system.

Figure 7a,b show the time-series data from the 15 sensors placed in an empty scanner-cast inside the empty MSR. Figure 7a shows data recorded over a 10-minute period with the dynamic nulling off, while Fig. 7b shows data recorded with dynamic nulling on. Vertical lines show the approximate times that a train arrived at either Russell Square or Holborn stations during the two different recording periods, thick lines show two trains arriving at the same time. During the nulling-off recording, 10 trains were reported in the 10-minute period, while for the nulling-on recording 7 trains were reported.

The maximum peak-to-peak variation of the time-series data across the 15 sensors was reduced from 2.37 nT to 0.13 nT by the dynamic nulling. The maximum standard deviation from the mean of the sensor outputs across sensors was reduced from 0.501 nT to 0.012 nT.

Figure 7c shows the median power spectral density estimate across all 15 sensors. To compute this estimate, the data were split into three non-overlapping sections of 200-s duration. For each section, flattop windowing (using the MATLAB function flattopwin) was applied prior to calculation of the power spectral density via fast Fourier transform. The results were then averaged over the three sections for each sensor and the median taken across all 15 sensors. No additional processing (e.g. forming synthetic gradiometers or linear regression) was applied to the raw magnetometer traces to remove other sources of interference. As a result, the noise spectra are mostly dominated by interference sources, which exceed the noise level specifications of the OPM sensors (https://quspin.com/products-qzfm/).

Figure 7d shows the shielding factor of the system computed as the ratio of the PSDs shown in Fig. 7c for the nulling-off and nulling-on cases. The shielding factor is reported in decibels (dB).

### Investigating interactions with the shielded room

**Figure 8:**
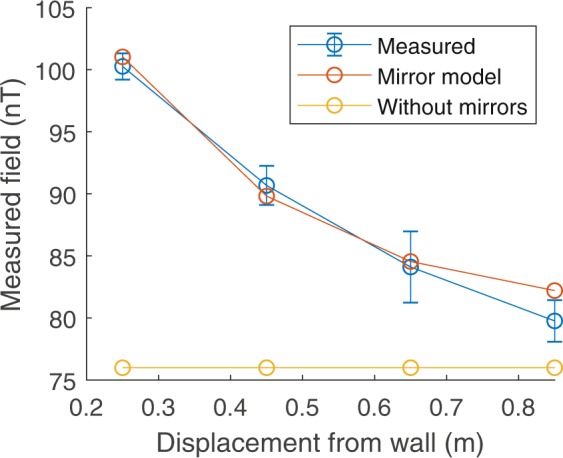
Experimental test of the mirroring approach to modelling coil interactions with high-permeability materials. The measured amplitude of a sinusoidally varying B_z_ magnetic field produced by a single coil is plotted as a function of displacement from the wall of the MSR. Simulated data both with and without the mirroring is provided for comparison.

Figure 8 shows the measured B_z_-field amplitude as a function of the z-displacement of the coil plane from the wall of the MSR, errorbars are calculated as the standard deviation of the amplitude over ten cycles. The expected enhancement of the measured field amplitude with proximity to the wall of the MSR is seen. Good agreement with the calculated data is also evident, indicating that the mirroring method provides a reasonable model of the interaction between the coils and the walls of the MSR.

**Figure 9:**
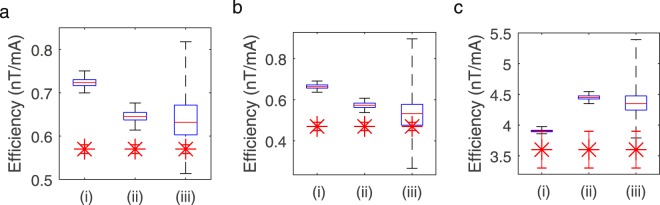
Investigating the effects of interactions of the coils with the mu-metal walls on the efficiency and homogeneity of the produced fields. (**a**–**c**) show for B_x_, B_y_ and B_z_ respectively: (i) a boxplot of the full range of fields calculated over a 40 × 40 × 40 cm^3^ grid when the coils are operating in free space, (ii) when the bi-planar coils are centred in the room and mirror currents up to third order are included and (iii) when the coils are positioned as they would be during an experiment. The red star shows the measured efficiency with standard deviation of the coils when setup for an experiment. Experimental values of B_x_ and B_y_ were averaged over three OPMs spatially separated in z at z = −8 cm, z = 0 cm and z = + 8 cm. For the B_z_ measure the sensors were positioned at z = −17 cm, z = 0 cm and z = + 17 cm for the B_z_ measurement.

Figure 9 shows box plots representing the mean and spatial variation over a central 40 × 40 × 40 cm^3^ volume of the calculated (A) B_x_, (B) B_y_ and (C) B_z_ fields per unit current (i.e. coil efficiencies) for three cases, where: (i) no mirror currents were considered; (ii) the bi-planar coils were assumed to be centrally positioned within the MSR and mirror currents up to third order were considered; (iii) the bi-planar coils were positioned off-centre, as they would be during an experiment, and mirror currents up to third order were considered. The measured experimental value of the efficiency and its associated standard deviation is shown as a red star with error bar for each case. As indicated previously, the experimental values of B_x_ B_y_ and B_z_ were averaged over three OPMs spatially separated in z. If the experimental value lies within the range of the box plots this is a good indication of the model being broadly accurate. High spatial homogeneity is desirable for producing a controlled field over the subject.

For the case of the B_x_ coil with no mirroring of currents, the median calculated field over the grid was 0.72 nT/mA with a range of 0.05 nT/mA (maximum value 0.7 nT/mA and minimum value 0.75 nT/mA). Positioning the coils at the centre of the MSR and adding mirror currents reduced the median calculated field to 0.64 nT/mA with a range of 0.06 nT/mA (max/min 0.61/0.67 nT/mA). Realistic positioning of the coils within the MSR and accounting for current mirroring further reduced the median efficiency to 0.62 nT/mA with a range of 0.21 nT/mA (max/min 0.67/0.46 nT/mA). The measured value of the efficiency of 0.57 ± 0.01 nT/mA lies within the increased range of field values. The simulations show that the field homogeneity decreases when the effect of the MSR is included. The maximum deviation of the field over the 40 × 40 × 40 cm^3^ volume that was considered increases from 5% with no consideration of mirror currents to 26% when the effects of the mirror currents are included for the realistically positioned coils.

Similar results were obtained for the B_y_ coil with the median simulated field decreasing from 0.66 nT/mA with a range of 0.05 nT/mA (max/min 0.64/0.69 nT/mA) when mirror currents were not considered to 0.54 nT/mA with a range of 0.17 nT/mA (max/min 0.6/0.43 nT/mA) when the coils were realistically positioned and the effects of the mirror currents were included. This range includes the measured value of 0.47 ± 0.02 nT/mA. The field inhomogeneity is increased from 5% to 22% for this coil when the mirror currents are included

In the case of the B_z_ coil, the median field increased from 3.90 nT/mA with a range of 0.11 nT/mA (max/min 3.97/3.86 nT/mA) to 4.27 nT/mA with a range of 0.61 nT/mA (max/min 4.45/3.84 nT/mA) on inclusion of mirror currents with realistic coil positioning. The upper bound of the error on the experimentally measured value of 3.60 ± 0.30 nT/mA lies within this range, but the general trend of an increased efficiency is not observed. The inhomogeneity is increased from 5% to 10% for this coil when the effect of the mirror currents is taken into account.

## Discussion

### Coil design and construction

By introducing a design and control method to produce coils which provide field nulling of six field terms using only three coil designs, construction of a field nulling system for use in wearable MEG is considerably simplified. The performance of the new field-nulling system is comparable to that obtained previously using a six-coil system^7^.

Although simpler designs such as the ‘Helmholtz-cage’ system described by Iivanainen and colleagues do not require complex wirepaths or use of large biplanar coils the subject has to remain fixed within a cubic enclosure^15^. A compromise has to be made upon system design between the freedom of motion and the quality of the MEG data which is obtained. Shielded rooms with larger remnant fields and gradients and larger temporally varying fields will pose a greater challenge for field nulling and as a result a fixed system may be simpler to implement. Additional compensation of higher order terms as well as improved dynamic nulling could allow for subject movement in poorer shielded environments.

To fully compensate for the uniform field and field gradients 8 terms (3 uniform fields and 5 gradients) are required, as the divergence and curl of the field are both zero in the current-free region between the planes. The system described here only produces 6 field terms. In this bi-planar arrangement the additional dB_x_/dy and dB_x_/dx terms have no shared symmetry with the uniform coils (or with one another) and would have to be individually constructed. Designs for these coils have been detailed previously, but they were not constructed^7^.

Although the number of coils that have to be wound is halved with this approach, the process of manual construction is still a cumbersome task and will likely result in errors such as inaccurate wire placement, misalignment from the wallpapering of the coils and warping of the wood. Although the PID approach allows for some of these errors to be taken into account, and the field nulling performs well, 3D printing, printed circuit board etching or water cutting techniques could potentially be used to increase the accuracy of coil winding and decrease labour requirements. With these techniques, the complexity of wire paths that could be produced would also increase.

### Static and dynamic field nulling

Though the system described here provides nulling which allows for movements by a seated subject, additional coils could also be constructed to provide better nulling and potentially allow a greater range of movements. These could include coils to generate the remaining two field gradients (dB_x_/dx and dB_x_/dy), individual, higher order spherical harmonics or more complex local patterns of field variation. We showed that dynamic nulling of uniform field components could be implemented using the balanced bi-planar coils, and this allowed the OPMs to be operated continually in a magnetically noisy environment, meaning that experiments could be completed without saturation of sensor outputs due to interference from a nearby underground railway line.

The relatively low mutual inductance (M) and self-inductance (L) of the coils (all <400 μH) and the low frequency of operation (<120 Hz) meant that the effects of inductance were not important when driving the coils since MdI/dt and LdI/dt were both always much smaller than IR (where I is the current and R is the coil resistance).

The slight increase in noise level which is introduced in the nulling-on case (see Fig. 7c,d) produces a negative shielding factor at some frequencies above 1 Hz. This noise results from the limited dynamic range of the field nulling electronics used here. To compensate for the remnant static field components the field nulling system has to be able to produce tens of nT; the NI-9264 DAC module has a ± 10 V dynamic range and a 16-bit resolution, the least significant bit is therefore equivalent to 20/2^16^ ~ 0.3 mV. In an example system, where 40 nT is required from 10 V this is equivalent to a field of roughly 1 pT. Rounding errors in setting the voltage in dynamic nulling consequently lead to additional field variation at the pT level at the sensor. In the low frequency range (<1 Hz) the environmental field variation is much larger than 1 pT and the dynamic nulling performs well, producing a significant shielding factor. However, at higher frequencies the environmental field noise is lower in magnitude and the additional field noise from the limited dynamic range of the nulling system becomes the dominating factor. This additional noise is apparent in the presented data, but as the effects are systematic and picked up by all sensors, synthetic gradiometry could be used to attenuate them.

To attenuate these effects in future field nulling systems a 24-bit DAC could be used to increase the dynamic range. The development of a two-circuit driver, one optimised for static nulling (producing e.g. 40 nT from 10 V) and one for dynamic nulling (producing e.g. 4 nT from 10 V) with the sum of the currents applied to the coils could be developed.

The speed of the LabVIEW based controller is fundamentally limited by the loop rate of the software. More advanced controllers, such as field programmable gate array microcontrollers or analogue solutions will be required for increased nulling performance at higher frequencies.

Although here the reference sensors were mounted in a scanner-cast during the MEG recording they would have to be placed behind the subject as shown in Fig. 3 in a real experiment, since with head-mounted reference sensors subject movements would reorient the reference sensors away from the x, y and z directions. This setup would allow the dynamic nulling to be readily expanded to include compensation of the field gradients G_x_, G_y_ and G_z_ through measurement of the full vector field on both sides of the head. This has not yet been implemented due to the limited number of sensors available for use in the reference array. A more complex arrangement of the reference array and feedback mechanism will most likely be required to provide an optimal dynamic null over an entire head array of OPMs. Flexible arrays, body mounted reference sensors and prior knowledge of the static magnetic field at different points combined with optical tracking could be used to provide a stable null as the subject moves within the MSR. Such improvements in the performance of coil technology for field compensation will be needed to facilitate new avenues of OPM-MEG research, such as experiments involving spatial navigation and direct social interaction between individuals.

### Interactions with the shielded room

Our brief investigation of the use of mirror currents to model interactions with the MSR revealed a change in median efficiency and reduction in homogeneity of the produced field which broadly agreed with the experimental measurements (Figs 8 and 9). The results imply the need to incorporate the effects of the high-permeability mu-metal into future coil designs, but it should be noted that the PID approach to both static and dynamic field nulling will seek to find the best solution without relying on a priori information.

Comparison of the boxplots in Fig. 9 shows that, for all three coils, the effect of the MSR on the field homogeneity is much less when the coils are positioned at the centre of the room. The off-centre positioning of the coil results in an asymmetric distribution of the additional field components from the mirror currents which make the field inhomogeneity larger than the ±5% condition targeted at the design stage. This asymmetry will also affect the efficiency and homogeneity of the gradient components, distorting the spatial distribution of the produced field. Unfortunately, the coils cannot be positioned centrally in the MSRs at UCL and Nottingham, as a ~500 kg cryogenic MEG system is sited at the centre of each room. Increased displacement of the coils from the centre of the MSR in the x and y directions would further effect the spatial distribution of the mirrored current elements; the closer a coil plane is to the wall, the greater the asymmetry of the resulting field distribution.

The experimental values were taken over single lines with a 16 cm spread for the B_x_ and B_y_ cases and a 34 cm spread for the B_z_ case instead of being measured over the entire 40 × 40 × 40 cm^3^ region. This may account for some of the discrepancy between experimental and theoretical measurements. In general, the interaction between the coils and the mu-metal walls should be more thoroughly investigated in order to incorporate the effect of the MSR when designing future coils. A more thorough experimental test of the range of validity of the simple mirror approach for static and dynamically-varying fields would be worthwhile, complemented by electromagnetic calculations using finite element analysis software.

## Supplementary information

Supplementary information

## Data Availability

The datasets generated during and/or analysed during the current study are available from the corresponding author on request.
