# Supplementary material for: Balanced, bi-planar magnetic field and field gradient coils for field compensation in wearable magnetoencephalography
Source: Sci Rep. 2019 Oct 2;9:14196. doi: 10.1038/s41598-019-50697-w (PMC6775070; doi:10.1038/s41598-019-50697-w)
Supplement: Supplementary file 1 — Supplementary information [file 41598_2019_50697_MOESM1_ESM.pdf]

Balanced, bi-planar magnetic field and field gradient coils for field compensation in wearable magnetoencephalography

Supplementary Information

Niall Holmes, Tim M Tierney, James Leggett, Elena Boto, Stephanie Mellor, Gillian Roberts, Ryan M Hill, Vishal Shah, Gareth R Barnes, Matthew J Brookes and Richard Bowtell

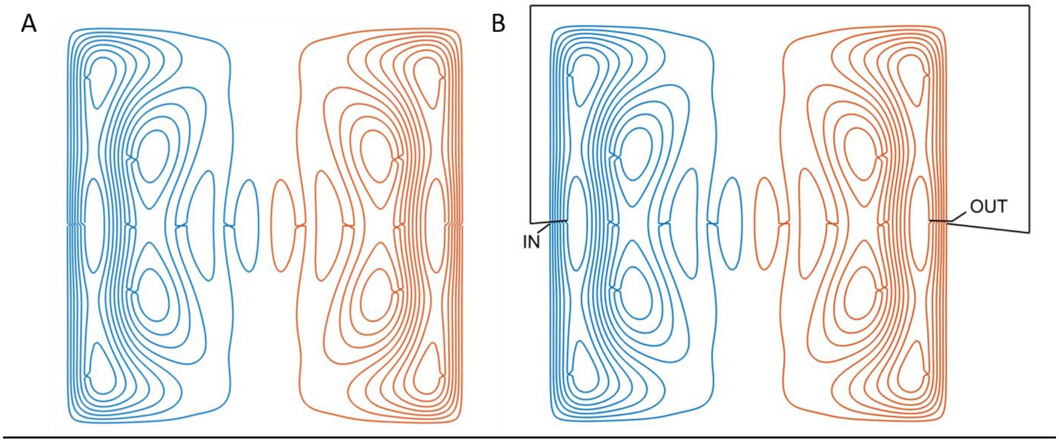

SI Figure 1: The connected wirepath for the B_x/G_x coil. (A) Contours were manually connected to provide a simple path with minimal cross-over points ensuring correct current circulation. This coil was wound in two halves. (B) The return connections were overlaid on the linking wires so that the additional magnetic fields produced by the linking wires were cancelled at a short distance from the coil planes. The two halves were then connected in series. To cancel the field produced from the series connection the wire was traced back from the 'OUT' point to the 'IN' point. The B_y/G_y coil was also wound in this manner.

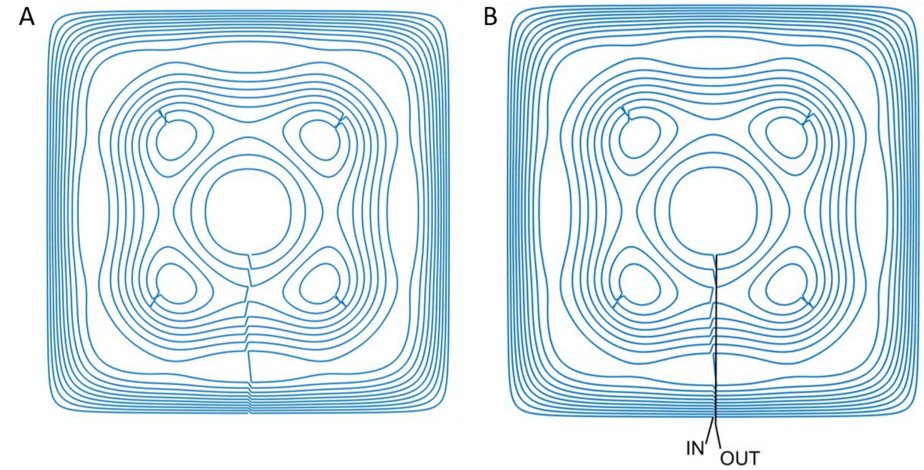

SI Figure 2: The connected wirepath for the B_z/G_z coil. (A) Contours were manually connected to provide a simple path with minimal cross-over points ensuring correct current circulation. This coil was wound as a continuous winding. (B) The return connections were overlaid on the linking wires so that the additional magnetic fields produced by the linking wires were cancelled at a short distance from the coil planes.
